# Supplementary material for: Investigation of superspreading COVID-19 outbreak events in meat and poultry processing plants in Germany: A cross-sectional study
Source: PLoS One. 2021 Jun 10;16(6):e0242456. doi: 10.1371/journal.pone.0242456 (PMC8191887; doi:10.1371/journal.pone.0242456)
Supplement: S1 Table — (DOCX) [file pone.0242456.s001.docx]

| Working area | Companies with this work area | Number of Employees overall | Overall,  without missing | Regular workers | Temporary and contract workers | Missing employment status | No. (%) of SARS-CoV-2 cases among workers |
| --- | --- | --- | --- | --- | --- | --- | --- |
| Delivery | 19 | 148 | 132 (100%) | 85 (64.4%) | 47 (35.6%) | 16 | 1 (0.7) |
| Anesthesia/ slinging/ hanging | 15 | 284 | 267 (100%) | 170 (63.7%) | 97 (36.3%) | 17 | 1 (0.4) |
| Slaughter | 15 | 1,259 | 1,099 (100%) | 200 (18.2%) | 899 (81.8%) | 160 | 71 (5.6) |
| Deboning and meat cutting area | 16 | 6,720 | 5,734 (100%) | 1,830 (31.9%) | 3,904 (68.1%) | 986 | 546 (8.1) |
| Meat production | 10 | 1,007 | 917 (100%) | 476 (51.9%) | 441 (48.1%) | 90 | 47 (4.7) |
| Sausage production | 7 | 489 | 291 (100%) | 152 (52.2%) | 139 (47.8%) | 198 | 6 (1.2) |
| Smoking of meat | 6 | 138 | 101 (100%) | 69 (68.3%) | 32 (31.7%) | 37 | 0 (0) |
| Packaging | 21 | 3,999 | 3,002 (100%) | 901 (30%) | 2,101 (70%) | 997 | 112 (2.8) |
| Commissioning/Loading | 21 | 948 | 850 (100%) | 627 (73.8%) | 223 (26.2%) | 98 | 24 (2.5) |
| Garage | 22 | 799 | 624 (100%) | 570 (91.7%) | 54 (8.7%) | 175 | 14 (1.8) |
| Cleaning of slaughter and production | 21 | 781 | 716 (100%) | 85 (11.9%) | 631 (88.1%) | 65 | 16 (2) |
| Administration | 22 | 1,254 | 946 (100%) | 926 (97.9%) | 20 (2.1%) | 308 | 14 (1.1) |
| Other work areas | 15 | 1,246 | 1,121 (100%) | 653 (58.3%) | 468 (42.7%) | 125 | 28 (2.2) |
| Overall | 22 | 19,072 | 15,800 (100%) | 6744 (42.7%) | 9056 (57.3%) | 3.272 | 880 (4.5) |
