# Supplementary material for: Investigation of superspreading COVID-19 outbreak events in meat and poultry processing plants in Germany: A cross-sectional study
Source: PLoS One. 2021 Jun 10;16(6):e0242456. doi: 10.1371/journal.pone.0242456 (PMC8191887; doi:10.1371/journal.pone.0242456)
Supplement: S2 Table — Abbreviations: OR, odds ratio; AOR, adjusted odds ratio; 95% CI, 95% confidence interval; OAF outdoor air flow. (DOCX) [file pone.0242456.s002.docx]

| Characteristics | Overall N=2,786/n*=2,154  without new OAF | | Without delivery, anesthesia/ slinging/hanging, slaughter n=2,334/n*=1,702 without new OAF | | With interaction term n=2,334/n*=1,702  without new OAF | |
| --- | --- | --- | --- | --- | --- | --- |
|  | OR (95% CI) | AOR (95% CI) | OR (95% CI) | AOR (95% CI) | OR (95% CI) | AOR (95% CI) |
| Maximal outdoor air flow (OAF) per employee m³/h | 1.000 (1.000-1.000) | 1.000 (1.000-1.000) | 0.994 (0.991-0.996) | 0.996 (0.993-0.999) | 0.959 (0.945-0.973) | 0.984 (0.971-0.996) |
| Maximal outdoor air flow (OAF) per employee m³/h without new OAF* | 1.000 (1.000-1.000) | 1.000 (1.000-1.000) | 0.995 (0.992-0.997) | 0.994 (0.990-0.998) | 0.971 (0.959-0.984) | 0.983 (0.969-0.997) |
| Interaction term temperature and OAF m³/h without new OAF* | - | - | - | - | 1.002 (1.001-1.004) | 1.001 (1.000-1.003) |
