# Supplementary material for: Investigation of superspreading COVID-19 outbreak events in meat and poultry processing plants in Germany: A cross-sectional study
Source: PLoS One. 2021 Jun 10;16(6):e0242456. doi: 10.1371/journal.pone.0242456 (PMC8191887; doi:10.1371/journal.pone.0242456)
Supplement: S1 Appendix — (PDF) [file pone.0242456.s003.pdf]

Fragebogen zur Evaluation der Schutzmaßnahmen gegen Neuinfektionen durch das Corona-Virus

Informationen zur Durchführung der Befragung

Möglicherweise müssen für einzelne Fragen verschiedene Ansprechpartner einbezogen werden.
Wenn diese keine exakten Angaben machen können, verdeutlichen Sie Ihren Ansprechpartnern, dass eine Annäherung bzw. Schätzung der tatsächlichen Zahl besser ist als keine Angabe.

Vielen Dank für Ihre Unterstützung!

A. Angaben zum Unternehmen bzw. der Betriebsstätte

Name des Unternehmens:	
Betriebsstätte:	
Adresse:	

B. Arbeitsbereiche/ Beschäftigte/ Infizierte

1. Arbeitsbereiche

1.1 Wie viele Beschäftigte arbeiten in den folgenden Arbeitsbereichen?

Wenn Sie keine genauen Angaben machen können, geben Sie bitte eine möglichst genaue Schätzung ab.

Arbeitsbereich	Anzahl der Beschäftigten	Anzahl der Schichten/Tag
**Anlieferung**		
**Betäubung/ Anschlingen/ Einhängen**		
**Schlachtung**		
Schwarzbereich		
Weißbereich		
**Zerlegung**		
Grobzerlegung		
Feinzerlegung		
**Fleischproduktion**		
**Wurstproduktion**		
**Räucherabteilung**		
**Verpackung**		
Weißbereich		
Schwarzbereich		
**Kommissionierung/ Verladung**		
**Werkstatt**		
**Reinigung Schlachtung/Produktion**		
**Verwaltung**		
**Sonstige**		

2. Art der Beschäftigungsverhältnisse

2.1 Wie viele Personen arbeiten in Ihrem Werk?

	Anzahl der Beschäftigten
**Stammpersonal**	
**Werkvertragsarbeitnehmer**	
**Leiharbeitnehmer**	

2.2 Gibt es Arbeitsbereiche, in denen keine Werkvertragsarbeitnehmer/ Leiharbeitnehmer beschäftigt sind?

◯	Nein	
◯	Ja, und zwar:	

2.3 Wie sind Werkvertragsarbeitnehmer/ Leiharbeitnehmer untergebracht?

	Geschätzter Anteil in Prozent
**Sammelunterkunft**	
**Gemeinschaftsunterkunft**	
**Mietwohnung, privates Mietverhältnis**	

2.4 Mit welchem Beförderungsmittel kommen die Werksarbeitnehmer/ Leiharbeitnehmer in den Betrieb (z.B. private Anreise / Kleinbus / Sammelbus / …)?

3. Infizierte Personen

3.1 Wie viele Infizierte sind ihnen bekannt und in welchen Arbeitsbereichen waren diese beschäftigt?

Arbeitsbereich	Anzahl der Infizierten
**Anlieferung**	
**Betäubung/ Anschlingen/ Einhängen**	
**Schlachtung**	
Schwarzbereich	
Weißbereich	
**Zerlegung**	
Grobzerlegung	
Feinzerlegung	
**Fleischproduktion**	
**Wurstproduktion**	
**Räucherabteilung**	
**Verpackung**	
Weißbereich	
Schwarzbereich	
**Kommissionierung/ Verladung**	
**Werkstatt**	
**Reinigung Schlachtung/Produktion**	
**Verwaltung**	
**Sonstige:**	

3.2 Wie viele infizierte Werkvertragsarbeitnehmer/Leiharbeiter wohnen in den folgenden Unterkunftsarten?

	Infizierte	Gesamtzahl
Sammelunterkunft		
Gemeinschaftsunterkunft		
Mietwohnung, privates Mietsverhältnis		

C. Umgebungsbedingungen/ Lüftung/ Klima

C1. Welche Arbeitsbereiche sind mit einer raumlufttechnischen Anlage (RLT) mit Außenluftzufuhr versehen?

Arbeitsbereich	RLT vorhanden	Außenluft-anteil in %	Umluft-anteil in %
**Anlieferung**	☐		
**Betäubung/ Anschlingen/ Einhängen**	☐		
**Schlachtung**	☐		
Schwarzbereich	☐		
Weißbereich	☐		
**Zerlegung**	☐		
Grobzerlegung	☐		
Feinzerlegung	☐		
**Fleischproduktion**	☐		
**Wurstproduktion**	☐		
**Räucherabteilung**	☐		
**Verpackung**	☐		
Weißbereich	☐		
Schwarzbereich	☐		
**Kommissionierung/ Verladung**	☐		
**Werkstatt**	☐		

Arbeitsbereich	RLT vorhanden	Außenluft-anteil in %	Umluft-anteil in %
Reinigung Schlachtung/Produktion	☐		
Verwaltung	☐		
Sonstige:			
	☐		
	☐		

C2. Welche Arbeitsbereiche werden auf welche Temperatur gekühlt?

Arbeitsbereich	Temperatur [°C]
**Anlieferung**	
**Betäubung/ Anschlingen/ Einhängen**	
**Schlachtung**	
Schwarzbereich	
Weißbereich	
**Zerlegung**	
Grobzerlegung	
Feinzerlegung	
**Fleischproduktion**	
**Wurstproduktion**	
**Räucherabteilung**	
**Verpackung**	
Weißbereich	
Schwarzbereich	
**Kommissionierung/ Verladung**	
**Werkstatt**	
**Reinigung Schlachtung/Produktion**	

Arbeitsbereich	Temperatur [°C]
**Verwaltung**	
**Sonstige:**	

C3. Wie hoch ist der maximale Außenluftanteil im gekühlten Arbeitsbereich im Sommer/ im Winter?

	Max. Außenluftanteil in %
**Sommersituation (hohe Temperaturen)**	
**Wintersituation (niedrige Temperatur)**	

C4. In welchen Arbeitsbereichen wird die Raumluft mit welchem Verfahren behandelt? (Filtration, UV-Strahlung, chemische Verfahren…o.ä.)

Arbeitsbereich	Behandlungsart
**Anlieferung**	
**Betäubung/ Anschlingen/ Einhängen**	
**Schlachtung**	
Schwarzbereich	
Weißbereich	
**Zerlegung**	
Grobzerlegung	
Feinzerlegung	
**Fleischproduktion**	
**Wurstproduktion**	
**Räucherabteilung**	
**Verpackung**	
Weißbereich	

Arbeitsbereich	Behandlungsart
Schwarzbereich	
**Kommissionierung/ Verladung**	
**Werkstatt**	
**Reinigung Schlachtung/Produktion**	
**Verwaltung**	
**Sonstige:**	

C5. Wie werden die folgenden Räume belüftet?

	RLT-Anlage	Natürliche Lüftung (Fenster, Türen)
**Kantine**	☐	☐
**Pausenräume**	☐	☐
**Umkleideräume**	☐	☐
**Sanitärräume**	☐	☐

D. Organisatorische und persönliche Schutzmaßnahmen

D1. Für welche Arbeitsbereiche gibt es feste Pausenzeiten?

Arbeitsbereich	Keine feste Pausen-zeit	Feste Pausen-zeit, zeitlich nicht versetzt	Feste Pausen-zeiten, zeitlich versetzt
**Anlieferung**	○	○	○
**Betäubung/ Anschlingen/ Einhängen**	○	○	○
**Schlachtung**	○	○	○
Schwarzbereich	○	○	○
Weißbereich	○	○	○
**Zerlegung**	○	○	○

Arbeitsbereich	Keine feste Pausen-zeit	Feste Pausen-zeit, zeitlich nicht versetzt	Feste Pausen-zeiten, zeitlich versetzt
Grobzerlegung	◌	◌	◌
Feinzerlegung	◌	◌	◌
**Fleischproduktion**	◌	◌	◌
**Wurstproduktion**	◌	◌	◌
**Räucherabteilung**	◌	◌	◌
**Verpackung**	◌	◌	◌
Weißbereich	◌	◌	◌
Schwarzbereich	◌	◌	◌
**Kommissionierung/ Verladung**	◌	◌	◌
**Werkstatt**	◌	◌	◌
**Reinigung Schlachtung/Produktion**	◌	◌	◌
**Verwaltung**	◌	◌	◌
**Sonstige:**			
	◌	◌	◌
	◌	◌	◌

D2. Wie werden die Abstandsregelungen (1,5 m) in den Pausenräumen/Kantine umgesetzt?

D3. In welchen Arbeitsbereichen wird der Mindestabstand von 1,5m nicht eingehalten?

Arbeitsbereich	Abstand mind. 1,5m	Abstand weniger als 1,5m	Wenn Abstand <1,5m: Welche Maßnahmen werden ergriffen?
**Anlieferung**	☐	☐	
**Betäubung/ Anschlingen/ Einhängen**	☐	☐	
**Schlachtung**	☐	☐	
Schwarzbereich	☐	☐	
Weißbereich	☐	☐	
**Zerlegung**	☐	☐	
Grobzerlegung	☐	☐	
Feinzerlegung	☐	☐	
**Fleischproduktion**	☐	☐	
**Wurstproduktion**	☐	☐	
**Räucherabteilung**	☐	☐	
**Verpackung**	☐	☐	

**Arbeitsbereich**	**Abstand mind. 1,5m**	**Abstand weniger als 1,5m**	**Wenn Abstand <1,5m: Welche Maßnahmen werden ergriffen?**
Weißbereich	☐	☐	
Schwarzbereich	☐	☐	
**Kommissionierung/ Verladung**	☐	☐	
**Werkstatt**	☐	☐	
**Reinigung Schlachtung/Produktion**	☐	☐	
**Verwaltung**	☐	☐	
**Sonstige:**			
	☐	☐	
	☐	☐	

D4. In welchen Arbeitsbereichen gab bzw. gibt es eine Tragepflicht für Mund-Nase-Bedeckung?

**Arbeitsbereich**	**Vor Corona**	**Nach Corona**
**Anlieferung**	☐	☐
**Betäubung/ Anschlingen/ Einhängen**	☐	☐
**Schlachtung**	☐	☐
Schwarzbereich	☐	☐
Weißbereich	☐	☐
**Zerlegung**	☐	☐

**Arbeitsbereich**	**Vor Corona**	**Nach Corona**
Grobzerlegung	☐	☐
Feinzerlegung	☐	☐
**Fleischproduktion**	☐	☐
**Wurstproduktion**	☐	☐
**Räucherabteilung**	☐	☐
**Verpackung**	☐	☐
Weißbereich	☐	☐
Schwarzbereich	☐	☐
**Kommissionierung/ Verladung**	☐	☐
**Werkstatt**	☐	☐
**Reinigung Schlachtung/Produktion**	☐	☐
**Verwaltung**	☐	☐
**Sonstige**		
	☐	☐
	☐	☐

D5. Welche Art von Mund-Nasen-Bedeckung wird aktuell getragen?

Medizinischer Mundschutz	☐
Einweg-Astro-Haube	☐
FFP2	☐
Sonstige, und zwar:	☐

D6. Beschreiben Sie das Konzept zur Wahrung des Abstandgebotes in Umkleideräumen. Wird es bereits umgesetzt?

D7. Gibt es zusätzliche Maßnahmen, um die Anzahl der Kontakte der Mitarbeiter ab dem Einlass am Werkstor herabzusetzen?

Bitte senden Sie den ausgefüllten Fragebogen an:

Isabel.Dienstbuehl@bgn.de

oder

Berufsgenossenschaft Nahrungsmittel und Gastgewerbe
Präventionsleitung
Isabel Dienstbühl
Dynamostr. 7-11
68165 Mannheim
