# Supplementary material for: Investigation of superspreading COVID-19 outbreak events in meat and poultry processing plants in Germany: A cross-sectional study
Source: PLoS One. 2021 Jun 10;16(6):e0242456. doi: 10.1371/journal.pone.0242456 (PMC8191887; doi:10.1371/journal.pone.0242456)
Supplement: S2 Appendix — (PDF) [file pone.0242456.s004.pdf]

Fragebogen zur Evaluation der Schutzmaßnahmen gegen Neuinfektionen durch das Corona-Virus (V3)

Informationen zur Durchführung der Befragung

Möglicherweise müssen für einzelne Fragen verschiedene Ansprechpartner einbezogen werden.
Wenn diese keine exakten Angaben machen können, verdeutlichen Sie Ihren Ansprechpartnern, dass eine Annäherung bzw. Schätzung der tatsächlichen Zahl besser ist als keine Angabe.

Vielen Dank für Ihre Unterstützung!

A. Angaben zum Unternehmen bzw. der Betriebsstätte

Name des Unternehmens:	
Betriebsstätte:	
Adresse:	

B. Arbeitsbereiche/ Beschäftigte/ Infizierte

B1. Arbeitsbereiche

B1.1 Wie viele Beschäftigte arbeiten in den folgenden Arbeitsbereichen?

Wenn Sie keine genauen Angaben machen können, geben Sie bitte eine möglichst genaue Schätzung ab.

Arbeitsbereich	Anzahl der Beschäftigten	…davon Stamm-personal	Anzahl der Schichten/Tag
**Anlieferung**			
**Betäubung/ Anschlingen/ Einhängen**			
**Schlachtung**			
Schwarzbereich			
Weißbereich			
**Zerlegung**			
Grobzerlegung			
Feinzerlegung			
**Fleischproduktion**			
**Wurstproduktion**			
**Räucherabteilung**			
**Verpackung**			
Weißbereich			
Schwarzbereich			
**Kommissionierung/ Verladung**			
**Werkstatt**			
**Reinigung Schlachtung/Produktion**			
**Verwaltung**			
**Sonstige**			
**SUMME BESCHÄFTIGTE:**			

B2. Art der Beschäftigungsverhältnisse

B2.1 Wie viele Personen arbeiten in Ihrem Werk?

Wenn Sie keine genauen Angaben machen können, geben Sie bitte eine möglichst genaue Schätzung ab.

	**Anzahl der Beschäftigten**
**Stammpersonal**	
**Werkvertragsarbeitnehmer**	
**Leiharbeitnehmer**	

B2.2 Gibt es Arbeitsbereiche, in denen keine Werkvertragsarbeitnehmer/ Leiharbeitnehmer beschäftigt sind?

○	Nein	
○	Ja, und zwar:	

B2.3 Wie sind Werkvertragsarbeitnehmer/ Leiharbeitnehmer untergebracht?

Wenn Sie keine genauen Angaben machen können, geben Sie bitte eine möglichst genaue Schätzung ab.

	**Anzahl der Beschäftigten**
**Sammelunterkunft**	
**Gemeinschaftsunterkunft**	
**Mietwohnung, privates Mietverhältnis**	

B2.4 Mit welchem Beförderungsmittel kommen die Werksarbeitnehmer/ Leiharbeitnehmer in den Betrieb (z.B. private Anreise / Kleinbus / Sammelbus / …)?

B3. Infizierte Personen

B3.1 An welchem Datum haben Sie zum ersten Mal von einem infizierten Mitarbeiter erfahren? Wie ist diese Information zu Ihnen gelangt?

B3.2 Welche Maßnahmen wurden ergriffen, nachdem bekannt wurde, dass es in ihrem Betrieb mind. einen mit SARS-CoV-2 infizierten Mitarbeiter gibt?

Sollten in Ihrem Unternehmen noch keine infizierten Mitarbeiter aufgetreten sein: Gibt es einen Maßnahmenplan und wenn ja, was sieht er vor?

kurzfristig: *innerhalb weniger Stunden bis zu sieben Tage, z.B. Quarantäne, Schließung von Produktionslinien/ Abteilungen*
langfristig: *innerhalb weniger Wochen umgesetzt, z.B. Reihentestungen, Nachrüsten von Lüftungsanlagen)*

Art der Maßnahme	kurzfristig umgesetzt	langfristig umgesetzt	geplant
	☐	☐	☐
	☐	☐	☐
	☐	☐	☐
	☐	☐	☐
	☐	☐	☐

B3.3 Wie viele Infizierte sind ihnen bekannt und in welchen Arbeitsbereichen waren diese beschäftigt?

Arbeitsbereich	Anzahl der Infizierten	…davon Stammpersonal
**Anlieferung**		
**Betäubung/ Anschlingen/ Einhängen**		
**Schlachtung**		
Schwarzbereich		
Weißbereich		
**Zerlegung**		

Arbeitsbereich	Anzahl der Infizierten	…davon Stammpersonal
Grobzerlegung		
Feinzerlegung		
**Fleischproduktion**		
**Wurstproduktion**		
**Räucherabteilung**		
**Verpackung**		
Weißbereich		
Schwarzbereich		
**Kommissionierung/ Verladung**		
**Werkstatt**		
**Reinigung Schlachtung/Produktion**		
**Verwaltung**		
**Sonstige:**		

B3.4 Wie viele infizierte Werkvertragsarbeitnehmer/Leiharbeiter wohnen in den folgenden Unterkunftsarten?

	Anzahl Infizierte
**Sammelunterkunft**	
**Gemeinschaftsunterkunft**	
**Mietwohnung, privates Mietsverhältnis**	

C. Umgebungsbedingungen/ Lüftung/ Klima

C1. Welche Arbeitsbereiche sind mit einer raumlufttechnischen Anlage (RLT) mit Außenluftzufuhr versehen?

Arbeitsbereich	RLT vorhanden	Außenluft-volumenstrom [m³/h]	Umluftführung möglich
**Anlieferung**	☐		☐
**Betäubung/ Anschlingen/ Einhängen**	☐		☐
**Schlachtung**	☐		☐
Schwarzbereich	☐		☐
Weißbereich	☐		☐
**Zerlegung**	☐		☐
Grobzerlegung	☐		☐
Feinzerlegung	☐		☐
**Fleischproduktion**	☐		☐
**Wurstproduktion**	☐		☐
**Räucherabteilung**	☐		☐
**Verpackung**	☐		☐
Weißbereich	☐		☐
Schwarzbereich	☐		☐
**Kommissionierung/ Verladung**	☐		☐
**Werkstatt**	☐		
**Reinigung Schlachtung/Produktion**	☐		
**Verwaltung**	☐		
**Sonstige:**			
	☐		
	☐		

C2. Welche Arbeitsbereiche werden auf welche Temperatur gekühlt?

Arbeitsbereich	Temperatur [°C]
**Anlieferung**	
**Betäubung/ Anschlingen/ Einhängen**	
**Schlachtung**	
Schwarzbereich	
Weißbereich	
**Zerlegung**	
Grobzerlegung	
Feinzerlegung	
**Fleischproduktion**	
**Wurstproduktion**	
**Räucherabteilung**	
**Verpackung**	
Weißbereich	
Schwarzbereich	
**Kommissionierung/ Verladung**	
**Werkstatt**	
**Reinigung Schlachtung/Produktion**	
**Verwaltung**	
**Sonstige:**	

C3. Wie wird die Kühlung erreicht?

Arbeitsbereich	Dezentrale Umluftkühlung (Sekundärluft)	Zentrale Kühlung der Außenluft über eine RLT-Anlage	
		ohne Umluftanteil	mit Umluftanteil
**Anlieferung**	☐	☐	☐
**Betäubung/ Anschlingen/ Einhängen**	☐	☐	☐
**Schlachtung**	☐	☐	☐
Schwarzbereich	☐	☐	☐
Weißbereich	☐	☐	☐
**Zerlegung**	☐	☐	☐
Grobzerlegung	☐	☐	☐
Feinzerlegung	☐	☐	☐
**Fleischproduktion**	☐	☐	☐
**Wurstproduktion**	☐	☐	☐
**Räucherabteilung**	☐	☐	☐
**Verpackung**	☐	☐	☐
Weißbereich	☐	☐	☐
Schwarzbereich	☐	☐	☐
**Kommissionierung/ Verladung**	☐	☐	☐
**Werkstatt**	☐	☐	☐
**Reinigung Schlachtung/Produktion**	☐	☐	☐
**Verwaltung**	☐	☐	☐
**Sonstige:**			
	☐	☐	☐
	☐	☐	☐

C4. Mit welcher Technik wird die gekühlte Umluft gereinigt?

Arbeitsbereich	Dezentrale Umluftkühlung (Sekundärluft)		Umluftluftanteil der RLT-Anlage		separate Umluft-entkeimung	
	Filtration (wenn ja, welche?)	UVC	Filtration (wenn ja, welche?)	UVC	Filtration (wenn ja, welche?)	UVC
**Zerlegung**	☐	☐	☐	☐	☐	☐
Grobzerlegung	☐	☐	☐	☐	☐	☐
Feinzerlegung	☐	☐	☐	☐	☐	☐
**Fleischproduktion**	☐	☐	☐	☐	☐	☐
**Wurstproduktion**	☐	☐	☐	☐	☐	☐
**Verpackung**	☐	☐	☐	☐	☐	☐
Weißbereich	☐	☐	☐	☐	☐	☐
Schwarzbereich	☐	☐	☐	☐	☐	☐

C5. Wie werden die folgenden Räume belüftet?

	RLT-Anlage	Natürliche Lüftung (Fenster, Türen)
**Kantine**	☐	☐
**Pausenräume**	☐	☐
**Umkleideräume**	☐	☐
**Sanitärräume**	☐	☐

D. Organisatorische und persönliche Schutzmaßnahmen

D1. Für welche Arbeitsbereiche gibt es feste Pausenzeiten?

**Arbeitsbereich**	**Keine feste Pausen-zeit**	**Feste Pausen-zeit, zeitlich nicht versetzt**	**Feste Pausen-zeiten, zeitlich versetzt**
**Anlieferung**	☐	☐	☐
**Betäubung/ Anschlingen/ Einhängen**	☐	☐	☐
**Schlachtung**	☐	☐	☐
Schwarzbereich	☐	☐	☐
Weißbereich	☐	☐	☐
**Zerlegung**	☐	☐	☐
Grobzerlegung	☐	☐	☐
Feinzerlegung	☐	☐	☐
**Fleischproduktion**	☐	☐	☐
**Wurstproduktion**	☐	☐	☐
**Räucherabteilung**	☐	☐	☐
**Verpackung**	☐	☐	☐
Weißbereich	☐	☐	☐
Schwarzbereich	☐	☐	☐
**Kommissionierung/ Verladung**	☐	☐	☐
**Werkstatt**	☐	☐	☐
**Reinigung Schlachtung/Produktion**	☐	☐	☐
**Verwaltung**	☐	☐	☐
**Sonstige:**			
	☐	☐	☐
	☐	☐	☐

D2. Wie werden die Abstandsregelungen (1,5 m) in den Pausenräumen/Kantine umgesetzt?

D3. In welchen Arbeitsbereichen wird der Mindestabstand von 1,5m nicht eingehalten?

Arbeitsbereich	Abstand mind. 1,5m	Abstand weniger als 1,5m	Wenn Abstand <1,5m: Welche Maßnahmen werden ergriffen?
**Anlieferung**	☐	☐	
**Betäubung/ Anschlingen/ Einhängen**	☐	☐	
**Schlachtung**	☐	☐	
Schwarzbereich	☐	☐	
Weißbereich	☐	☐	
**Zerlegung**	☐	☐	
Grobzerlegung	☐	☐	
Feinzerlegung	☐	☐	

Arbeitsbereich	Abstand mind. 1,5m	Abstand weniger als 1,5m	Wenn Abstand <1,5m: Welche Maßnahmen werden ergriffen?
**Fleischproduktion**	☐	☐	
**Wurstproduktion**	☐	☐	
**Räucherabteilung**	☐	☐	
**Verpackung**	☐	☐	
Weißbereich	☐	☐	
Schwarzbereich	☐	☐	
**Kommissionierung/ Verladung**	☐	☐	
**Werkstatt**	☐	☐	
**Reinigung Schlachtung/Produktion**	☐	☐	
**Verwaltung**	☐	☐	
**Sonstige:**			
	☐	☐	
	☐	☐	

D4. In welchen Arbeitsbereichen gab bzw. gibt es eine Tragepflicht für Mund-Nase-Bedeckung?

Arbeitsbereich	Vor Corona	Nach Corona
**Anlieferung**	☐	☐
**Betäubung/ Anschlingen/ Einhängen**	☐	☐
**Schlachtung**	☐	☐
Schwarzbereich	☐	☐
Weißbereich	☐	☐
**Zerlegung**	☐	☐
Grobzerlegung	☐	☐
Feinzerlegung	☐	☐
**Fleischproduktion**	☐	☐
**Wurstproduktion**	☐	☐
**Räucherabteilung**	☐	☐
**Verpackung**	☐	☐
Weißbereich	☐	☐
Schwarzbereich	☐	☐
**Kommissionierung/ Verladung**	☐	☐
**Werkstatt**	☐	☐
**Reinigung Schlachtung/Produktion**	☐	☐
**Verwaltung**	☐	☐
**Sonstige**		
	☐	☐
	☐	☐

D5. Welche Art von Mund-Nasen-Bedeckung wird aktuell getragen?

Medizinischer Mundschutz	☐
Einweg-Astro-Haube	☐
FFP2	☐
Sonstige, und zwar:	☐

D6. Beschreiben Sie das Konzept zur Wahrung des Abstandgebotes in Umkleideräumen. Wird es bereits umgesetzt?

D7. Gibt es zusätzliche Maßnahmen, um die Anzahl der Kontakte der Mitarbeiter ab dem Einlass am Werkstor herabzusetzen?

Bitte senden Sie den ausgefüllten Fragebogen an:

Isabel.Dienstbuehl@bgn.de

oder

Berufsgenossenschaft Nahrungsmittel und Gastgewerbe
Präventionsleitung
Isabel Dienstbühl
Dynamostr. 7-11
68165 Mannheim
