# Supplementary material for: Investigation of superspreading COVID-19 outbreak events in meat and poultry processing plants in Germany: A cross-sectional study
Source: PLoS One. 2021 Jun 10;16(6):e0242456. doi: 10.1371/journal.pone.0242456 (PMC8191887; doi:10.1371/journal.pone.0242456)
Supplement: S3 Appendix — (PDF) [file pone.0242456.s005.pdf]

Questionnaire for the evaluation of protective measures against new infections caused by the Corona virus (V3)

Information on how to conduct the survey

Different contact persons may need to be involved for individual questions.
If they cannot provide exact information, tell your contacts that an approximation or estimate of the actual number is better than no indication.

Thank you for your support!

A. Information about the company or the plant

name of the company:	
plant:	
address:	

B. Working areas/ employees/ infected people

B1. Working areas

B1.1 How many employees work in following working areas?

If you are unable to provide precise information, please provide the most accurate estimate possible.

Working area	Number of employees overall	… of which are contract workers	Number of shifts per day
**Delivery**			
**Anesthesia/ slinging/ hanging**			
**Slaughter**			
Black area			
White area			
**Deboning and meat cutting area**			
Coarse deboning and cutting			
Fine deboning and cutting			
**Meat production**			
**Sausage production**			
**Smoking of meat**			
**Packaging**			
White area			
Black area			
**Commissioning/ Loading**			
**Garage**			
**Cleaning of slaughter and production**			
**Administration**			
**Other work areas**			
**Sum of the employees:**			

B2. Type of employment

B2.1 How many people work in your company?

If you are unable to provide precise information, please provide the most accurate estimate possible.

	Number of employees
**Regular workers**	
**Contract workers**	
**Temporary workers**	

B2.2 Are there any areas of work where there are no contract workers?

○	No	
○	Yes, and that is:	

B2.3 How are contract workers/temporary workers accommodated?

If you are unable to provide precise information, please provide the most accurate estimate possible.

	Number of employees
**Collective accommodation**	
**Shared accommodation**	
**Flat, private lease**	

B2.4 What means of transport do the factory workers /temporary workers use to enter the company (f.e. private arrival / minibus / collective bus / ...)?

B3. Infected people

B3.1 What date did you first hear about an infected employee? How did this information come to you?

B3.2 What measures were taken after it became known that there is at least one employee infected with SARS-CoV-2 in the company?

If your company has not yet had any infected employees, is there a plan of action and, if so, what does it provide for?

Implemented short term: *within a few hours or up to 7 days, f.e. quarantine, closure of production lines/departments*
Implemented long term: *within a few weeks implemented, f.e. series testing, retrofit ventilation systems)*

Type of protective measure	Implemented short term	Implemented long term	planned
	☐	☐	☐
	☐	☐	☐
	☐	☐	☐
	☐	☐	☐
	☐	☐	☐

B3.3 How many infected people are known to the company and in which working areas were they employed?

Working area	Number of infected people	… of which are contract workers
**Delivery**		
**Anesthesia/ slinging/ hanging**		
**Slaughter**		
Black area		
White area		

**Working area**	**Number of infected people**	**… of which are contract workers**
**Deboning and meat cutting area**		
Coarse deboning and cutting		
Fine deboning and cutting		
**Meat production**		
**Sausage production**		
**Smoking of meat**		
**Packaging**		
White area		
Black area		
**Commissioning/ Loading**		
**Garage**		
**Cleaning of slaughter and production**		
**Administration**		
**Other work areas:**		

B3.4 How many infected contract/ temporary workers stay in the following types of accommodation?

	**Number of infected people**
**Collective accommodation**	
**Shared accommodation**	
**Flat, private lease**	

C. Working environment/ ventilation/ air condition

C1. Which working areas are equipped with a ventilation system with an outdoor air supply?

Working area	ventilation system implemented?	outdoor air flow [m³/h]	air circulation possible?
**Delivery**	☐		☐
**Anesthesia/ slinging/ hanging**	☐		☐
**Slaughter**	☐		☐
Black area	☐		☐
White area	☐		☐
**Deboning and meat cutting area**	☐		☐
Coarse deboning and cutting	☐		☐
Fine deboning and cutting	☐		☐
**Meat production**	☐		☐
**Sausage production**	☐		☐
**Smoking of meat**	☐		☐
**Packaging**	☐		☐
White area	☐		☐
Black area	☐		☐
**Commissioning/ Loading**	☐		☐
**Garage**	☐		
**Cleaning of slaughter and production**	☐		
**Administration**	☐		
**Other work areas:**			
	☐		
	☐		

C2. Which working areas are cooled to which temperature?

Working area	Temperature [°C]
**Delivery**	
**Anesthesia/ slinging/ hanging**	
**Slaughter**	
Black area	
White area	
**Deboning and meat cutting area**	
Coarse deboning and cutting	
Fine deboning and cutting	
**Meat production**	
**Sausage production**	
**Smoking of meat**	
**Packaging**	
White area	
Black area	
**Commissioning/ Loading**	
**Garage**	
**Cleaning of slaughter and production**	
**Administration**	
**Other work areas:**	

C3. How is the cooling achieved?

Working area	Decentralized cooled circulating air (Secondary air)	Central cooling of the outdoor air with a ventilation system	
		without cooled circulating air	with cooled circulating air
**Delivery**	☐	☐	☐
**Anesthesia/ slinging/ hanging**	☐	☐	☐
**Slaughter**	☐	☐	☐
Black area	☐	☐	☐
White area	☐	☐	☐
**Deboning and meat cutting area**	☐	☐	☐
Coarse deboning and cutting	☐	☐	☐
Fine deboning and cutting	☐	☐	☐
**Meat production**	☐	☐	☐
**Sausage production**	☐	☐	☐
**Smoking of meat**	☐	☐	☐
**Packaging**	☐	☐	☐
White area	☐	☐	☐
Black area	☐	☐	☐
**Commissioning/ Loading**	☐	☐	☐
**Garage**	☐	☐	☐
**Cleaning of slaughter and production**	☐	☐	☐
**Administration**	☐	☐	☐
**Other work area:**			
	☐	☐	☐

Working area	Decentralized cooled circulating air (Secondary air)	Central cooling of the outdoor air with a ventilation system	
		without cooled circulating air	with cooled circulating air
	☐	☐	☐

C4. What technology is used to clean the cooled circulating air?

Working area	Decentralized cooled circulating air (Secondary air)		cooled circulating air of the ventilation system		separate circulating air disinfection	
	Filtration (if so, which?)	UVC	Filtration (if so, which?)	UVC	Filtration (if so, which?)	UVC
**Deboning and meat cutting area**	☐	☐	☐	☐	☐	☐
Coarse deboning and cutting	☐	☐	☐	☐	☐	☐
Fine deboning and cutting	☐	☐	☐	☐	☐	☐
**Meat production**	☐	☐	☐	☐	☐	☐
**Sausage production**	☐	☐	☐	☐	☐	☐
**Packaging**	☐	☐	☐	☐	☐	☐
White area	☐	☐	☐	☐	☐	☐
Black area	☐	☐	☐	☐	☐	☐

C5. How are the following rooms ventilated?

	ventilation system	Natural ventilation (windows, doors)

**Canteen**	☐	☐
**Break rooms**	☐	☐
**Changing rooms**	☐	☐
**Sanitary rooms**	☐	☐

D. Organizational and personal protective measures

D1. For which work areas are there fixed break times?

Working area	No fixed break time	Fixed break time, not time shifted	Fixed break time, time shifted
**Delivery**	☐	☐	☐
**Anesthesia/ slinging/ hanging**	☐	☐	☐
**Slaughter**	☐	☐	☐
Black area	☐	☐	☐
White area	☐	☐	☐
**Deboning and meat cutting area**	☐	☐	☐
Coarse deboning and cutting	☐	☐	☐
Fine deboning and cutting	☐	☐	☐
**Meat production**	☐	☐	☐
**Sausage production**	☐	☐	☐
**Smoking of meat**	☐	☐	☐
**Packaging**	☐	☐	☐
White area	☐	☐	☐
Black area	☐	☐	☐
**Commissioning/ Loading**	☐	☐	☐
**Garage**	☐	☐	☐
**Cleaning of slaughter and production**	☐	☐	☐
**Administration**	☐	☐	☐
**Other work areas:**			
	☐	☐	☐
	☐	☐	☐

D2. How are the distance controls (1.5 m) implemented in the break rooms/canteen?

D3. In which working areas the minimum distance of 1,5m is not observed?

Working area	Distance min. 1,5m	Distance less than 1,5m	If distance <1,5m: Which protective measure are being taken?
**Delivery**	☐	☐	
**Anesthesia/ slinging/ hanging**	☐	☐	
**Slaughter**	☐	☐	
Black area	☐	☐	
White area	☐	☐	
**Deboning and meat cutting area**	☐	☐	
Coarse deboning and cutting	☐	☐	
Fine deboning and cutting	☐	☐	
**Meat production**	☐	☐	

Working area	Distance min. 1,5m	Distance less than 1,5m	If distance <1,5m: Which protective measure are being taken?
**Sausage production**	☐	☐	
**Smoking of meat**	☐	☐	
**Packaging**	☐	☐	
White area	☐	☐	
Black area	☐	☐	
**Commissioning/ Loading**	☐	☐	
**Garage**	☐	☐	
**Cleaning of slaughter and production**	☐	☐	
**Administration**	☐	☐	
**Other work areas:**			
	☐	☐	
	☐	☐	

D4. In which working areas do the company has an obligation to carry a mouth-nose cover?

Working areas	Before Corona	After Corona
**Delivery**	☐	☐
**Anesthesia/ slinging/ hanging**	☐	☐
**Slaughter**	☐	☐
Black area	☐	☐
White area	☐	☐
**Deboning and meat cutting area**	☐	☐
Coarse deboning and cutting	☐	☐
Fine deboning and cutting	☐	☐
**Meat production**	☐	☐
**Sausage production**	☐	☐
**Smoking of meat**	☐	☐
**Packaging**	☐	☐
White area	☐	☐
Black area	☐	☐
**Commissioning/ Loading**	☐	☐
**Garage**	☐	☐
**Cleaning of slaughter and production**	☐	☐
**Administration**	☐	☐
**Other work areas:**		
	☐	☐
	☐	☐

D5. What kind of face covering is currently worn?

Medical face covering	☐
Astronaut cap with face mask	☐
FFP2 mask	☐
Other:	☐

D6. Describe the concept of maintaining distance in changing rooms. Is it already being implemented?

D7. Are there any additional measures to reduce the number of contacts between employees at the factory entrance?

Please send the completed questionnaire to following address:

Isabel.Dienstbuehl@bgn.de

or

Berufsgenossenschaft Nahrungsmittel und Gastgewerbe
Präventionsleitung
Isabel Dienstbühl
Dynamostr. 7-11
68165 Mannheim
