# Supplementary material for: Investigation of superspreading COVID-19 outbreak events in meat and poultry processing plants in Germany: A cross-sectional study
Source: PLoS One. 2021 Jun 10;16(6):e0242456. doi: 10.1371/journal.pone.0242456 (PMC8191887; doi:10.1371/journal.pone.0242456)
Supplement: Attachment — Submitted filename: Reponese to reviewers_20210326.docx [file pone.0242456.s006.docx]

**Reviewer comments in black font, Author replies in black font italic and underlined.**

**Comments to the Author**

1. Is the manuscript technically sound, and do the data support the conclusions?

The manuscript must describe a technically sound piece of scientific research with data that supports the conclusions. Experiments must have been conducted rigorously, with appropriate controls, replication, and sample sizes. The conclusions must be drawn appropriately based on the data presented.

Reviewer #1: Yes

2. Has the statistical analysis been performed appropriately and rigorously?

Reviewer #1: Yes

3. Have the authors made all data underlying the findings in their manuscript fully available?

The [PLOS Data policy](http://www.plosone.org/static/policies.action#sharing) requires authors to make all data underlying the findings described in their manuscript fully available without restriction, with rare exception (please refer to the Data Availability Statement in the manuscript PDF file). The data should be provided as part of the manuscript or its supporting information, or deposited to a public repository. For example, in addition to summary statistics, the data points behind means, medians and variance measures should be available. If there are restrictions on publicly sharing data—e.g. participant privacy or use of data from a third party—those must be specified.

Reviewer #1: Yes

4. Is the manuscript presented in an intelligible fashion and written in standard English?

PLOS ONE does not copyedit accepted manuscripts, so the language in submitted articles must be clear, correct, and unambiguous. Any typographical or grammatical errors should be corrected at revision, so please note any specific errors here.

Reviewer #1: Yes

5. Review Comments to the Author

Please use the space provided to explain your answers to the questions above. You may also include additional comments for the author, including concerns about dual publication, research ethics, or publication ethics. (Please upload your review as an attachment if it exceeds 20,000 characters)

Reviewer #1: The authors have conducted a cross-sectional analysis of meat processing plants to assess factors associated with risk for transmission of COVID-19. They surveyed 26 plants and included 7 companies with more than 10 cases in their analyses. The results identified lack of social distancing, cool temperature and lack of air exchange as factors associated with higher rates of transmission. These findings are consistent with other reports and provide companies with guidance for reducing the risk of transmission in their facilities. While useful, there are several issues that need to be addressed.
The survey identified plants with a range of infection rates. There was no comparison to identify differences between plants with high infection rates versus low infection rates. It seems that this could have been useful, and there should be some comment about why this wasn’t pursued, or if it was pursued, why it wasn’t reported.
Line 61-64. It is not clear how contract workers mentioned here relate to the temporary or contract workers who comprised the main study population. It is somewhat confusing throughout to understand the differences between permanent, contract and temporary workers. Please elaborate.

*We have clarified our wording and the problems with the wording. After the evaluation of the first version of the questionnaire we decided to combine contract and temporary workers.* *The definition and the exact difference of the two groups of temporary and contract workers is not selective and we tried to clarify our use in the method section and added a section in the method part in line 141ff.*

Lines 110-111 The distribution of companies by cases is based on the interval June-September?

*Yes, data was collected between June and September 2020. Earliest outbreak by a participant plant was May 2020 and was the first outbreak in a meat processing facility in Germany.*

Line 112 What is “paper and pencil questionnaire”? Is this just to distinguish from an online survey?

*Since the companies were given a word document to fill out, we changed this word to “questionnaire”.*

Line 142 “point prevalence” seems really to be more of a period-based incidence measure.

*We agree with the reviewer that the wording is here a little bit problematic. The best description is maybe to describe each outbreak as a “point prevalence” and the combined prevalence from all companies a “period-based prevalence”. We clarified that in the manuscript.*

Line 147 Why were there no comparisons between plants with different categories of infection?

*We analyzed the differences across plants with different categories of infection. The comparison is unfortunately a little bit problematic because we had to analyze free text and put it in categories. Also, the occupational safety measures display safety measures at different time points. Now we included some intervention and prevention efforts of the facilities (line 234ff) and put a new section in the method part as well in the discussion section.*

Line 150 Is the difference between fixed breaks with or with time shifts a reflection on whether every takes a break at the same time or not? This could be clarified.

*Yes. We clarified that and added an explanation: “Fixed breaks without time shift mean that all workers have their breaks at the same time. Fixed breaks with time shift mean that there is a break pattern where different working groups, departments or divisions have different time windows for their break.”*

Line 162 The calculation assumes that all shifts have the same number of employees. Is that correct?

*Yes, the assumption is that each shift in a company has the same number of employees. Like we wrote in the discussion the visits in the companies confirmed our assumption about the actual number of potentially exposed subjects during a shift.*

Lines 191-194 Are these calculations manipulations of the model to predict outcomes for defined exposure levels? This warrants a bit more description.

*Exactly. These calculations are based on the same model and only the amount of fresh air has been changed. We excluded this from the manuscript, because it does not add any further knowledge to the association of the variables.*

Line 210 What does it mean that one plant took part although it was not contacted?

*It means that the parent company has distributed the questionnaire to all of its companies and all companies of the parent company have answered the questionnaire. Therefore, we received one questionnaire from a company we did not write to.*

Table 1 Why were employees excluded?

*Employees were excluded when information was missing. We discussed the possibility of imputation, but because our analysis was only possible because working areas in the meat processing area are clearly distinct, we decided that we don’t want to impute missing values, for example for the cleaning personal. Our procedure is explained at the end of the statistical analysis part.*

In Table 1 there is a column heading for contract workers. In Table 3 the corresponding column headings mentions regular workers. These appear to be the same and should be called the same.

*We agree and clarified our wording into regular, contract and temporary workers. Thank you for this advice.*
